# Understanding the Financial Implications of Antimicrobial Resistance Surveillance in Nepal: Context-Specific Evidence for Policy and Sustainable Financing Strategies

**DOI:** 10.3390/antibiotics15010103

**Published:** 2026-01-20

**Authors:** Yunjin Yum, Monika Karki, Dan Whitaker, Kshitij Karki, Ratnaa Shakya, Hari Prasad Kattel, Amrit Saud, Vishan Gajmer, Pankaj Chaudhary, Shrija Thapa, Rakchya Amatya, Timothy Worth, Claudia Parry, Wongyeong Choi, Clemence Nohe, Adrienne Chattoe-Brown, Deepak C. Bajracharya, Krishna Prasad Rai, Sangita Sharma, Kiran Pandey, Bijaya Kumar Shrestha, Runa Jha, Jung-Seok Lee

**Affiliations:** 1 International Vaccine Institute, Seoul 08826, Republic of Korea; yunjin.yum@ivi.int (Y.Y.); wongyeong.choi@ivi.int (W.C.); clemence.nohe@ivi.int (C.N.); 2 G.T.A. Foundation, Lalitpur 44700, Nepal; monika.karki1031@gmail.com (M.K.); kshitij@gtanepal.org (K.K.); shrija@gtanepal.org (S.T.); rakchya@gtanepal.org (R.A.); deepak@gtanepal.org (D.C.B.); 3 Network of International Consultants in Health and Development (NICHAD), London NW1 9LP, UK; dan.whitaker@nichad.org; 4 Mott MacDonald Ltd., London EC4M 7RB, UK; timothy.worth@mottmac.com (T.W.); claudia.parry@mottmac.com (C.P.); adrienne.chattoe-brown@mottmac.com (A.C.-B.); 5 National Food and Feed Reference Laboratory, Department of Food Technology and Quality Control, Kathmandu 44600, Nepal; shakya.tamrakar@gmail.com (R.S.); krishnaprdrai@gmail.com (K.P.R.); 6 Tribhuvan University Teaching Hospital, Kathmandu 44600, Nepal; harikattel@iom.edu.np (H.P.K.); drsangita_sharma@iom.edu.np (S.S.); 7 Veterinary Laboratory, Pokhara 33700, Nepal; amritsaud172@gmail.com (A.S.); kiranpandey5@gmail.com (K.P.); 8 National Avian Disease Investigation Laboratory, Bharatpur 44200, Nepal; vishan.gajmer@nepal.gov.np (V.G.); bijaya.shrestha@nepal.gov.np (B.K.S.); 9 Koshi Hospital, Biratnagar 56700, Nepal; pankajchy1987@gmail.com (P.C.); runa75jha@gmail.com (R.J.)

**Keywords:** antimicrobial resistance (AMR), AMR surveillance, AMR surveillance cost, micro-costing, sustainable financing, Nepal

## Abstract

**Background/Objectives**: Antimicrobial resistance (AMR) surveillance is a cornerstone of national AMR strategies but requires sustained, cross-sectoral financing. While the need for such financing is well recognized, its quantification remains scarce in low- and middle-income countries. This study aimed to estimate the full costs of AMR surveillance across the human health, animal health, and food sectors (2021–2030) in selected facilities in Nepal and generate evidence to inform sustainable financing. **Methods**: A bottom-up micro-costing approach was used to analyze data from five sites. Costs were adjusted for inflation using projected gross domestic product deflators, and probabilistic sensitivity analyses were conducted to assess uncertainty in laboratory sample volumes under four scenarios. **Results**: The total cost of AMR surveillance in Nepal was $6.7 million: $3.4 million for human health (50.3% out of the aggregated costs), $2.7 million for animal health (39.8%), and $0.7 million for the food sector (9.9%). Laboratories accounted for >90% of total costs, with consumables and personnel as the main cost drivers. Average cost per sample was $150 (animal), $64 (food), and $6 (human). **Conclusions**: This study offers the first robust, multi-sectoral 10-year cost estimates of AMR surveillance in Nepal. The findings highlight that sustaining AMR surveillance requires predictable domestic financing, particularly to cover recurrent laboratory operations as donor support declines. These results provide cost evidence to support future budgeting and policy planning toward sustainable, nationally financed AMR surveillance in Nepal.

## 1. Introduction

Antimicrobial resistance (AMR) has emerged as a major threat to global public health, diminishing the effectiveness of antimicrobial agents in controlling infections and leading to prolonged hospital stays, higher treatment failure rates, and increased healthcare costs [1,2,3]. It is estimated that AMR causes around 25,000 deaths annually in Europe, imposing an economic burden of approximately €1.5 billion through healthcare costs and productivity losses [4]. In the United States, AMR is associated with an estimated 23,000 deaths each year, alongside $20 billion in direct medical expenses and $35 billion due to productivity losses [4]. Beyond direct impacts, the macroeconomic consequences are substantial: the World Bank projects that, in the absence of effective action, AMR could reduce global gross domestic product (GDP) by 3.8% by 2050, with an even larger decline (4.4%) in low- and middle-income countries (LMICs) [5].

The impact of AMR is particularly critical in LMICs, where infectious disease incidence is higher, and health systems are less equipped to respond [4,5,6]. In these settings, AMR is driven by a combination of inappropriate antibiotic use, weak or poorly enforced legislation, and limited access to quality healthcare and antibiotics due to high out-of-pocket costs and supply shortages. In the animal health sector, the use of antibiotics as growth promoters in livestock production and limited awareness of good management practices, along with antimicrobial residues entering the food chain, further contribute to the emergence and spread of resistance [7,8,9,10,11]. Strengthening AMR surveillance systems is therefore essential for detecting emerging resistance patterns and guiding timely interventions [12,13]. Otherwise, AMR will easily spread across borders. However, surveillance is resource-intensive, requiring investments in infrastructure, laboratory capacity, trained personnel, and ongoing operational costs. These demands pose a considerable challenge for LMICs. Evidence on the costs involved—across laboratory setup, operations, and multi-sectoral surveillance—remains very limited, making it difficult to advocate for national investments in AMR surveillance and to highlight its role in safeguarding public health and reducing the economic burden of AMR.

In resource-constrained countries such as Nepal, the financial burden of AMR surveillance may be substantial, given the considerable health burden caused by AMR and other competing health problems. In 2019, around 6400 deaths were attributable to AMR, and 23,200 deaths were associated with AMR in Nepal. Based on the age-standardized mortality rate per 100,000 population associated with AMR, Nepal ranked 52nd among 204 countries included in the global burden of AMR analysis [14]. Yet, the financial requirements for sustaining AMR surveillance activities—particularly beyond the human health sector—remain largely unknown [15].

The current study aims to estimate the costs of AMR surveillance in Nepal across the human health, animal health, and food sectors, addressing a critical evidence gap and providing data to inform sustainable financing strategies.

## 2. Results

Table 1 summarizes the AMR surveillance costs by sector and cost component. The first three years (2021–2023) and the subsequent seven years (2024–2030) were defined to capture the initial costs and later cost periods. From 2021 to 2030, the aggregated cost of AMR surveillance in Nepal was estimated to be $6,696,058 across five facilities. By sector, human health accounted for the largest share of total costs (50.3% out of the aggregated costs), followed by animal health (39.8%), and the food sector (9.9%) (Table 1). Cost breakdowns by component for each site are presented in Table S1.

**Figure 1:**
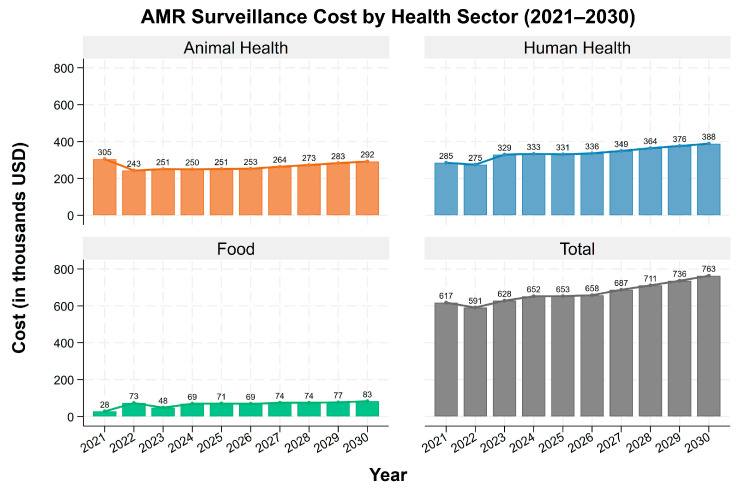
Antimicrobial resistance surveillance cost by sector.

Annual costs in the animal and human health sectors generally followed the aggregate trend, showing a decline in 2022 and an upward trend since 2022, with minor fluctuations (Figure 1). The food sector, however, showed a different trend, with the costs increasing sharply in 2022, falling in 2023, and then showing a slight upward trend while remaining relatively stable thereafter. These annual fluctuations can be explained by the changes in specific cost components. For example, in animal health, the decline in 2022 was driven by reduced spending on other direct costs and equipment, while in human health, it was primarily associated with lower expenditure on consumables. In the food sector, the increase in 2022 was due to substantial expenditure on other direct costs and equipment, while the subsequent decline in 2023 reflects reduced spending after these major purchases.

**Figure 2:**
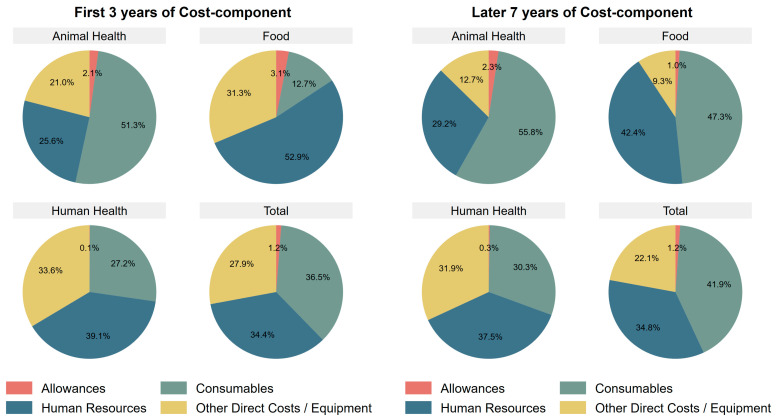
AMR surveillance cost by sector and component.

Figure 2 shows the distribution of costs by component. In the food sector, the relative contribution of cost components shifted over time. During the first three years (2021–2023), the expenditures on other direct costs and equipment were approximately three times those of consumables, whereas in the subsequent seven years (2024–2030), consumables accounted for the larger share. This likely reflects the high equipment purchase costs during 2021–2023, whereas large pieces of equipment are expected to last for several years, with subsequent expenditures mainly related to maintenance and utilities under other direct costs. In contrast, in the animal and human health sectors, the proportional contribution of cost components remained relatively stable overall, with a 3–5% increase in consumables for both sectors. Across all sectors, the proportion of the costs associated with other direct costs/equipment went down from the first three years to the following seven years, although the difference was marginal in the human health sector. Overall, in the later years, human resources and consumables represented the dominant cost components across the sectors.

Laboratory costs accounted for more than 90% of the total AMR surveillance cost. The average laboratory costs for the first three years by sector were $364,133 for animal health, $440,656 for human health, and $130,051 for food. The average cost per sample for the first three years was highest in the animal health sector ($149), followed by the food sector ($47) and the human health sector ($5), reflecting large differences in the total number of samples across sectors (Table 2).

**Figure 3:**
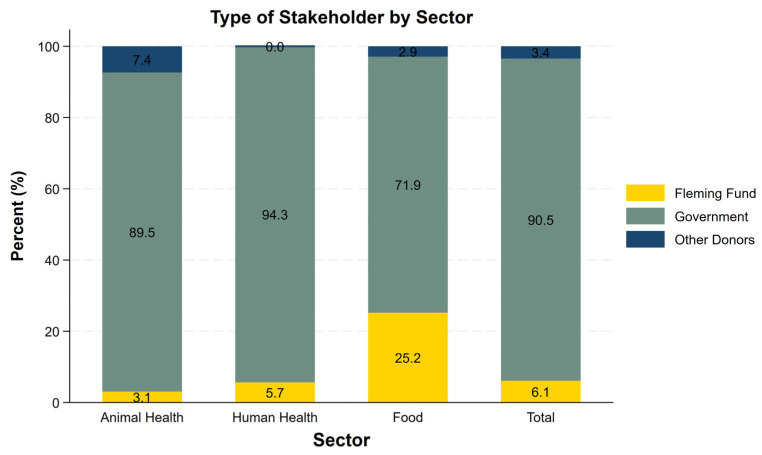
Type of stakeholder by sector for the first three years.

Figure 3 presents the distribution of financial contributions by stakeholders during the first three years of surveillance. Overall, the government accounted for the largest share (90.4%), followed by the Fleming Fund (6.1%) and other donors (3.4%). Across all sectors, government funding was the largest contributor. In the human health and food sectors, this was followed by the Fleming Fund, whereas in the animal health sector, other donors were the second largest contributors. The government’s share was particularly high in the human (94.3%) and animal health (89.6%) sectors compared to the food sector (71.9%). Figure 2 and Figure 3 together show that the animal and human health sectors maintain more stable cost-component structures and rely more on domestic financing, indicating that their AMR surveillance systems operate with greater autonomy and stability than those in the food sector.

**Figure 4:**
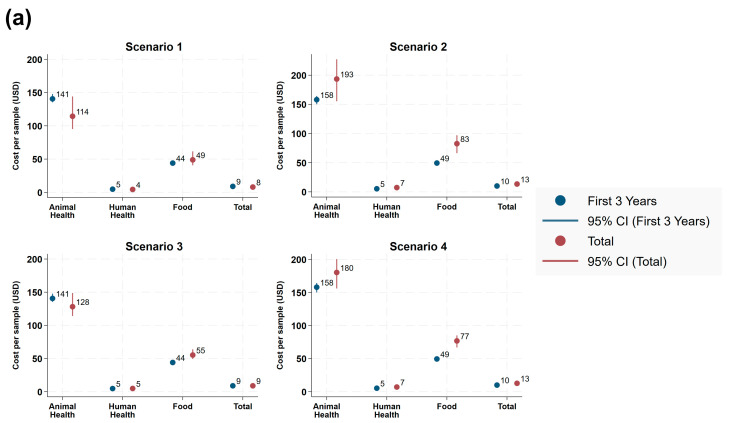

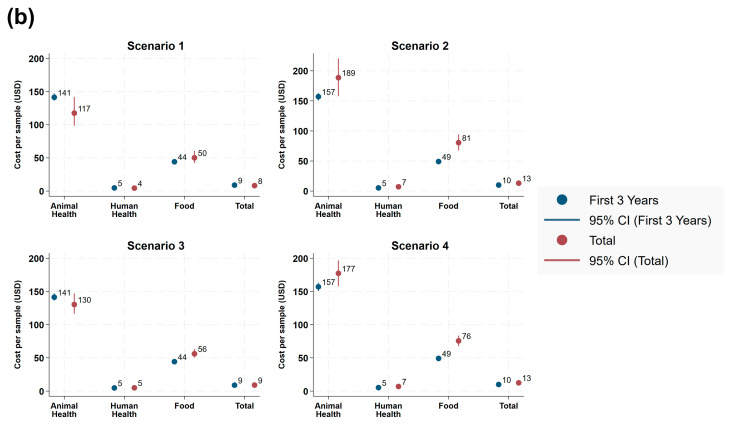
PSA results for antimicrobial resistance surveillance costs across sectors under four scenarios, based on (**a**) beta distribution (α = 1.47, β = 1.10) and (**b**) triangular distribution (a = 0, b = 0.1, c = 0.06). Equipment purchased at TUTH before 2021 may lead to an additional potential cost of approximately $19,988 if replaced (initial purchase cost, without inflation adjustment).

The probabilistic sensitivity analysis (PSA) results based on both the beta and triangular distributions showed consistent trends across scenarios. Under the beta distribution, the total costs were estimated to be $7.9 (6.6–10.0), $13.4 (10.8–15.8), $8.9 (7.9–10.5), and $12.8 (11.0–14.3) for scenarios 1–4, respectively (Figure 4). Under the triangular distribution, the total costs were estimated to be $8.2 (6.8–9.9), $13.1 (11.0–15.3), $9.1 (8.1–10.3), and $12.6 (11.1–14.0), respectively.

In scenarios 1 and 3, where the number of samples increased, the laboratory cost per sample for each health sector was the same or lower than under the assumption of constant sample volumes (Table 2). In scenario 3, even though the additional resources associated with the laboratory costs increased alongside increased sample volumes, the cost per sample remained the same or lower than that of the constant sample volumes. In contrast, scenarios 2 and 4, which assumed decreasing sample volumes, showed a higher cost per sample than the constant sample volumes. Overall, the PSA suggested that costs per sample appear to be influenced by sample volumes: higher volumes may reduce cost per sample through economies of scale.

## 3. Discussion

The current study provides one of the first comprehensive estimates of the costs of AMR surveillance across the human health, animal health, and food sectors in Nepal, using the micro-costing approach. By quantifying expenditures from 2021 to 2030 by sector, activity, and cost-component, the main cost drivers and evidence to inform future budget planning were identified. These findings can help prioritize critical components of the surveillance system to optimize resource allocations and support the development of sustainable financing strategies—particularly important in resource-constrained LMIC settings where competing health priorities limit available funding.

Our findings highlight three key messages. First, our study provides the first robust, multi-sectoral estimate of the total cost of AMR surveillance in Nepal over 10 years, amounting to $6.7 million. To date, no such comprehensive cost estimation has been undertaken in Nepal, or indeed anywhere else. While a few studies in other countries have examined AMR surveillance costs, they have typically been limited in scope. For example, a recent study in Timor–Leste evaluated the cost-effectiveness of maintaining an active hospital microbiology laboratory and demonstrated that such services can be cost-saving by reducing intensive care unit admissions and mortality. However, the analysis focused on a single tertiary hospital in the human health sector, and no full system costs of AMR surveillance across sectors were estimated [16]. Similarly, a study in Southeast Asian hospitals (Laos, Cambodia, and Thailand) estimated the cost of setting up and running a microbiology laboratory in the human health sector [17], but costs for other sectors and activities were not included. Moreover, several cost components, such as utilities, were not measured directly from each country but instead extrapolated from the figures reported elsewhere [17]. The essential non-lab costs of a national AMR surveillance system were also ignored. These categories are particularly important for long-term sustainability, as they represent recurrent expenditures that must be consistently funded to ensure the continuity and reliability of surveillance. Such costs across the human health, animal health, and food sectors were directly addressed in the current study, thereby filling a critical evidence gap and providing a strong reference point for sustainable financing.

Second, our analysis revealed distinct sectoral cost patterns and drivers. The human health sector accounted for the largest share of total costs, as AMR surveillance in the human health sector functions as an essential and prioritized clinical diagnostic activity. This also reflects a more advanced system and implementation, requiring higher personnel costs, specialized laboratory infrastructure, and larger sample volumes. The relatively lower costs observed in the food sector are expected to increase as the number of surveillance sites and activities expands in the future. Annual trends also differed: in the early years (2021–2022), the human and animal health sectors showed a decreasing trend, whereas the food sector showed an increase. The decline observed in the human sector was mainly due to an increase in sample size and normalization after a temporary reduction in 2022, which had resulted from the use of stockpiled consumables and the impact of the COVID-19 (Coronavirus Disease 2019) pandemic. The animal health and food sectors, where active AMR surveillance activities began only in recent years, were affected by changes in other direct costs and equipment-related costs. The decrease in the animal health sector and the increase in the food sector during 2021–2022 may partly reflect differences in the timing of AMR surveillance initiation, indicating more recent initiation in the food sector. Importantly, the differences in cost-component contributions between the early and later years indicate a transition toward higher recurrent costs over time. The relative contributions of other direct costs and equipment decreased in the later years compared with the initial years, whereas recurrent costs—particularly those related to human resources and consumables—became more prominent over time. This underscores the long-term financial challenge of sustaining AMR surveillance in LMICs, where recurrent expenditures must be financed once initial donor-funded setup investments taper off, often without clear national plans for long-term funding sources.

Beyond the sector-level findings, the current study paid special attention to the granularity of the estimates. By disaggregating costs by activity, this study provides findings that can inform specific policy actions—for example, targeting resources to data collection/sharing, external quality assurance, or surveys—rather than focusing solely on aggregate sectoral expenditures. In addition, identifying stakeholders who supported particular activities can help flag areas that may require donor support. For instance, high-cost procurement and associated maintenance, as well as many reagents and consumables for high-ticket items such as blood culture machines, are likely to fall into this donor-supported category. Such detailed evidence is particularly valuable in resource-constrained settings where prioritization of high-impact activities is essential.

Third, the laboratory-related cost dominated the surveillance budget, accounting for over 90% of the total expenditure. This finding is consistent with previous studies in Kenya, which also highlighted laboratories as the central cost driver of AMR surveillance systems [4]. Similar findings have been reported in Rwanda, where establishing basic microbiology diagnostic capacity required substantial laboratory-related investments [18]. In our study, average laboratory costs for 2021–2023 amounted to $146,885 in human health, $121,378 in animal health, and $43,350 in food, and per-sample costs are $5, $149, and $47, respectively. Notably, the large difference in cost per sample between the human and the other two sectors may partially be due to differences in sample volume, suggesting that routine microbiological testing may be conducted less frequently in the animal health and food sector compared with the human health sector. These site-level estimates and per-sample estimates capture the financial burden of establishing and operating individual laboratories, highlighting the need to strengthen routine testing capacity in these sectors. Together, these results underscore not only the dominance of laboratory expenditures but also their critical implications for sustainable financing and resource allocation. In addition, our probabilistic sensitivity analysis suggests that the increase in sample volume may help partially offset proportional rises in other cost components, indicating that maintaining sufficient sample throughput is important for cost-efficient surveillance. This pattern is consistent with recent cost-effectiveness evidence from Timor-Leste, which showed that maintaining an active microbiology laboratory can be cost-saving when sufficient sample volumes are achieved [16].

Sustaining AMR surveillance in Nepal will require predictable financing, particularly for high-cost equipment replacement and recurrent costs such as consumables and staff salaries. The observed cost structure suggests that the government and donor investments should not only focus on initial setup (e.g., laboratory equipment) but also ensure long-term operational funding, especially in sectors with low sample throughput and high fixed costs.

These findings should be interpreted in light of certain limitations. First, some cost data and future activities were unavailable and had to be estimated based on expert opinions at the facility-level. Although these were flagged in the costing tool, reliance on expert judgment may introduce uncertainty. As facilities acquire more complete data, these inputs can be updated; however, the assumptions and the representativeness of the current estimates should be considered when interpreting the results. Second, as data collection focused on the facility level, certain activities undertaken at higher administrative levels —such as national policy development, coordination meetings, and activities related to the National Action Plan—may not have been fully captured. As a result, the total cost of AMR surveillance at the national level may be underestimated. Third, future cost projections were adjusted using GDP deflators projected via ordinary least squares (OLS) regression. While OLS offers a transparent and interpretable method, it does not capture potential macroeconomic shocks, meaning actual future costs could deviate from projections. Future research could therefore consider alternative projection frameworks such as IMF (International Monetary Fund)-based projections or more sophisticated time-series models (e.g., Autoregressive Integrated Moving Average (ARIMA)) to better capture macroeconomic variability and enhance projection accuracy. Fourth, the study covers five sentinel sites; while these were coordinated by the Ministry of Health and Population (MOHP) of Nepal to represent national surveillance activities, they may not adequately capture the full range of operational contexts present in Nepal. In particular, the relatively high government contribution observed may be specific to the five included facilities, whereas other facilities that are more self-sufficient may show different cost structures. Inclusion of additional sites in future analyses would improve representativeness. Accordingly, cost-per-laboratory and cost-per-sample estimates should be interpreted with caution, as they may vary substantially across different facility types and levels of AMR surveillance activities. Lastly, this study considered cost data for the period 2021–2030 only; therefore, expenditures incurred outside this period were not captured. For example, equipment purchased before 2021 was not included in the analysis; thus, these data mostly represent ongoing costs rather than the total cost of setting up a surveillance system. Further interpretations should take into account the potential replacement of high-cost equipment, which has an estimated lifespan of ten years.

Despite these limitations, this study offers one of the most comprehensive, cross-sectoral cost estimates of AMR surveillance in a low-resource setting. By generating sector- and activity-level cost data, it provides critical evidence for Nepal’s policymakers to strengthen AMR containment strategies and negotiate sustainable financing with both government and external partners. More importantly, the methodological approach and key findings are also relevant for other LMICs facing similar challenges. In particular, our results illustrate the value of disaggregated cost data for informing national action plans, prioritizing scarce resources, and engaging donors in realistic discussions about long-term sustainability and autonomous planning. Ultimately, such evidence can support Nepal and other LMICs in transitioning from donor-dependent systems toward autonomous, nationally sustained AMR surveillance. Future research should extend this work by assessing the cost-effectiveness and health impact of AMR surveillance investments, integrating epidemiological outcomes with cost data to capture their broader societal value.

## 4. Materials and Methods

### 4.1. Micro-Costing Approach

To estimate the costs associated with AMR surveillance activities, a micro-costing (bottom-up) approach was applied. The approach comprised the following steps. First, two general lists of AMR surveillance activities were created to assist users unfamiliar with micro-costing, which are provided in Supplementary Files S1 and S2. The first list was drawn from Phase 1 of the Fleming Fund Country Grants projects in Nepal, Laos, and Uganda, identifying AMR surveillance activities by health sector and categorizing them into four cost components: human resources, allowances, consumables, and other direct costs/equipment. Human resources include personnel involved in the activities; allowances cover per diem or transportation costs related to travel and field duties; consumables include laboratory consumables, publishing, and other operational supplies; and other direct costs/equipment encompass equipment purchase, maintenance, and utility costs. The second list, based on a literature (desk) review at the global-level, summarized activities and sub-activities across various health sectors and country income levels classified by the World Bank. Second, all micro-level activities conducted at each site were identified, along with the specific resources required for each activity. Third, the quantity of each resource used was measured in natural units (e.g., staff time in days, number of consumable items, and number of equipment units). Finally, the cost of each activity was calculated by multiplying the quantity of each resource by its corresponding unit cost [19], allowing not only the estimation of current expenditures but also projections of future costs based on anticipated resource use. Using this approach allows costs to be estimated in a more detailed and precise manner, as the majority of resources involved in AMR surveillance can be classified as direct costs [20].

### 4.2. Excel-Based Costing Tool

To carry out this approach, an Excel-based costing tool that enabled systematic collection and estimation of AMR surveillance costs across the sectors was developed. The tool comprised six main components: a Cover sheet (providing background, study objectives, and purpose of the tool); an Input parameters sheet (for exchange rates, implicit deflators, stakeholder types, and site details); an Input costs & counts sheet (identifying AMR surveillance activity lists, cost categories, stakeholder information, unit costs, and counts of resource use); an Analysis sheet (annual cost summaries by stakeholder type, cost-component, and solidity classification); a Summary sheet (presenting outputs such as cost per stakeholder, cost per laboratory, and cost per sample); and an Appendices sheet (containing GDP deflator calculations for future years). Once the three input sheets (Input parameters, Input costs & counts, and Appendices) are completed, the tool automatically generates the summarized costs by category in the Analysis and Summary sheets.

### 4.3. Data Sources and Study Sites

Data on costs associated with AMR surveillance activities were obtained from site records, including finance department documents (e.g., salaries and utilities), inventory and stock registers, and external market price sources, from 2021 to 2024. When exact site-level values were unavailable, missing activity counts or unit costs were imputed using information from staff interviews, expert input from personnel involved in AMR surveillance implementation at each site (e.g., laboratory in-charges, finance officers, veterinary or investigation officers), or data from comparable sites. This approach was adopted because cost data may vary depending on institutional roles, laboratory functions, and the scope of diagnostic testing performed, even within the same health sectors. These entries were flagged in the “Solidity” column of the costing tool to indicate the strength of the underlying evidence and distinguish estimates or assumptions from values derived from exact records. Where feasible, values were cross-validated across sources and across sites. For example, unit costs and quantities for commonly procured antibiotics were compared across facilities, and procurement information for donor-funded equipment was cross-checked against both site records and available funder documentation. Counts from 2025 to 2030 were projected by assessing whether each cost item would remain necessary to sustain AMR surveillance activities without ongoing donor support.

**Figure 5:**
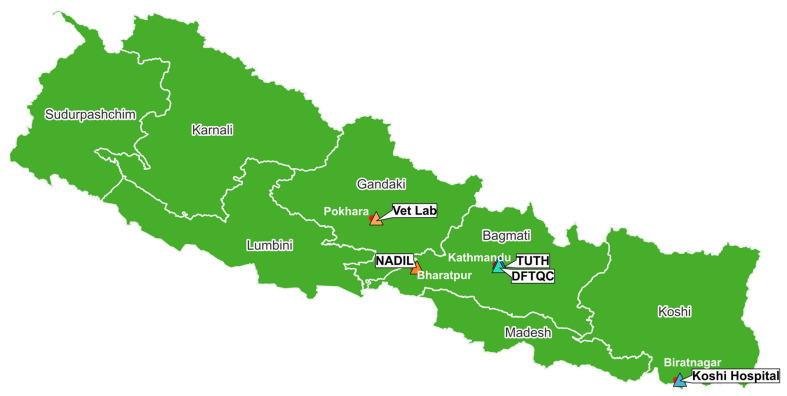
Locations of five study sites in Nepal. Red dots indicate the cities in which the study sites are located.

To estimate the costs of AMR surveillance in Nepal, five sites were selected across three sectors: 2 sites for animal health, 2 sites for human health, and 1 site for food (Table 3 and Figure 5). Site selection was coordinated by MOHP, and all five selected sites were Fleming Fund-supported AMR surveillance sites. For the animal and human health sectors, the National Technical Working Committee designated the National Public Health Laboratory (NPHL) and the Central Veterinary Laboratory (CVL) to identify eligible sites. NPHL selected two human health sites, and CVL, with approval from the Department of Livestock Services, selected two animal health sites for the study. In the animal health sector, two sentinel sites were included: the National Avian Disease Investigation Laboratory (NADIL, Bharatpur), one of the largest non-private poultry disease diagnostic centers, and the Veterinary Laboratory (Vet Lab, Pokhara), a government facility supporting livestock, poultry, and companion animal health [21,22]. In the human health sector, Koshi Hospital (Biratnagar, 350-bed government hospital) [23] and Tribhuvan University Teaching Hospital (TUTH, Kathmandu, 700-bed national referral teaching hospital) were chosen. For the food sector, data were collected from the Department of Food Technology and Quality Control (DFTQC, Kathmandu), the national reference laboratory responsible for food and quality standards. Table 3 summarizes key site information, including the average annual number of laboratory samples for microbiological examinations and utility costs for five sites.

### 4.4. Statistical Analysis

Statistical analyses were conducted to project AMR surveillance costs and to assess uncertainty in the future estimates. Inflation-adjusted costs were estimated using projected GDP deflators derived from OLS regression models. Uncertainty in future sample volumes and associated costs was evaluated using probabilistic sensitivity analysis. Monte Carlo simulation was used to generate point estimates and corresponding 95% confidence intervals. All analyses were performed using Excel 16.0 and Stata 18.0.

#### 4.4.1. Inflation Adjustment

As the unit cost presented the price in the year when they were incurred, future costs were adjusted for inflation. To do this, GDP deflator values were projected using an OLS regression based on 20 years of historical GDP deflator data obtained from the World Bank [24,25]. The projected values were then used to calculate the implicit deflators, which were applied to adjust unit costs over time. The OLS regression approach was chosen as it offers a transparent and straightforward method for projecting deflators from historical data.

#### 4.4.2. Sensitivity Analysis

To account for a degree of uncertainty in the future laboratory sample volumes and associated costs, PSA was conducted. In the main analysis, the annual number of samples was assumed to be constant across years; therefore, variation was introduced by applying annual increases or decreases in the number of samples and proportional changes in relevant resources drawn from probability distributions. A beta distribution, commonly used for probabilities or proportions, and a triangular distribution, suitable when empirical evidence is limited, were adopted [26,27,28]. Distributional parameters were derived from the inpatient admissions at a tertiary hospital in Nepal (not part of the study sites), used as a proxy for potential changes in sample volumes.

Four scenarios were specified. In scenarios 1 and 2, only the number of samples was assumed to either increase (scenario 1) or decrease (scenario 2) annually. On the other hand, the proportional change in human resources, consumables, and other direct costs and equipment was taken into account together with the change in the number of laboratory samples for scenarios 3 and 4, where the respective resources were assumed to increase in scenario 3 and decrease in scenario 4. These scenarios reflect the expected relationship, where increases or decreases in sample volume are typically accompanied by proportional changes in personnel needs, consumable use, and utility and maintenance expenses. Human resources, consumables, and other direct costs and equipment were referenced to their 2024 levels. Consumables were assumed to increase or decrease in proportion to laboratory sample volumes, while other cost components were assumed to change gradually at one-third of the sample growth rate. A summary of these four scenarios is provided in Table S2.

## 5. Conclusions

This study provides the first robust, multi-sectoral estimate of the 10-year (2021–2030) costs of AMR surveillance in Nepal, covering the human health, animal health, and food sectors across five sites. The total cost was estimated at $6.7 million. Laboratory-related activities accounted for over 90% of expenditures, and large differences in cost per sample, mainly due to the number of samples, highlight the importance of maintaining adequate sample volumes for cost-efficient surveillance. Cost structures further indicate a shift from early capital equipment expenditures toward recurrent operational costs, particularly human resources and consumables. This underscores the need for predictable domestic financing as donor support declines. Despite uncertainties in projections, these sector- and activity-level estimates provide critical evidence to support prioritization, budgeting, and sustainable financing of AMR surveillance in Nepal and other LMICs.

## Figures and Tables

**Table 1:** AMR surveillance costs by sector and component across the initial (2021–2023) and later (2024–2030) periods.

Sector	Cost-Component	First 3 Years (2021–2023)	Later 7 Years (2024–2030)	Total
Animal Health	Total	$798,782	$1,865,538	$2,664,319
	Human resources	$204,728 (25.6%)	$544,146 (29.2%)	$748,873 (28.1%)
	Allowances	$17,087 (2.1%)	$44,114 (2.4%)	$61,202 (2.3%)
	Consumables	$409,320 (51.2%)	$1,041,164 (55.8%)	$1,450,485 (54.4%)
	Other Direct Costs/Equipment	$167,646 (21.0%)	$236,114 (12.7%)	$403,760 (15.2%)
Human Health	Total	$889,108	$2,478,340	$3,367,448
	Human resources	$348,050 (39.1%)	$929,655 (37.5%)	$1,277,704 (37.9%)
	Allowances	$857 (0.1%)	$6714 (0.3%)	$7571 (0.2%)
	Consumables	$241,697 (27.2%)	$751,330 (30.3%)	$993,027 (29.5%)
	Other Direct Costs/Equipment	$298,506 (33.6%)	$790,640 (31.9%)	$1,089,146 (32.3%)
Food	Total	$148,290	$516,002	$664,291
	Human resources	$78,511 (52.9%)	$218,639 (42.4%)	$297,149 (44.7%)
	Allowances	$4536 (3.1%)	$5261 (1.0%)	$9797 (1.5%)
	Consumables	$18,778 (12.7%)	$244,098 (47.3%)	$262,875 (39.6%)
	Other Direct Costs/Equipment	$46,466 (31.3%)	$48,004 (9.3%)	$94,469 (14.2%)
Total		$1,836,179	$4,859,879	$6,696,058

Percentages indicate each component’s share of the sector total.

**Table 2:** AMR surveillance cost per laboratory and per sample by sector.

Sector	Site	First 3 Years (2021–2023)	Total (2021–2030)	Annually for the First 3 Years
Animal Health	NADIL	$461,999	$1,494,587	$154,000
	Cost per sample (n = 1050)	$147	$142	-
	Vet Lab	$266,268	$944,881	$88,756
	Cost per sample (n = 580)	$153	$163	-
	Total	$728,267	$2,439,468	$242,756
	Cost per sample	$149	$150	-
	Cost per lab	$364,133	$1,219,734	$121,378
Human Health	Koshi Hospital	$180,601	$763,234	$60,200
	Cost per sample (n = 8293)	$7	$9	-
	TUTH	$700,712	$2,570,950	$233,571
	Cost per sample (n = 50,372)	$5	$5	-
	Total	$881,312	$3,334,184	$293,771
	Cost per sample	$5	$6	-
	Cost per lab	$440,656	$1,667,092	$146,885
Food	DFTQC	$130,051	$595,097	$43,350
	Cost per sample (n = 931)	$47	$64	-
	Cost per lab	$130,051	$595,097	$43,350
Total	Cost per lab	$347,926	$1,273,750	$115,975
	Cost per sample	$9	$10	-

Abbreviations: NADIL: National Avian Disease Investigation Laboratory; Vet Lab: Veterinary Laboratory; TUTH: Tribhuvan University Teaching Hospital; DFTQC: Department of Food Technology and Quality Control.

**Table 3:** Site information.

Sector	Site	Location	Sample/Year	Utility Costs per Year
Animal Health	NADIL	Bharatpur	1050	$5387
	Vet Lab	Pokhara	580	$4685
Human Health	Koshi Hospital	Biratnagar	8293	$2425
	TUTH	Kathmandu	50,372	$94,724
Food	DFTQC	Kathmandu	931	$5625

Abbreviations: NADIL: National Avian Disease Investigation Laboratory; Vet Lab: Veterinary Laboratory; TUTH: Tribhuvan University Teaching Hospital; DFTQC: Department of Food Technology and Quality Control.

## Data Availability

Data supporting this article are available from the corresponding author upon reasonable request.

## Supplementary Materials

The following supporting information can be downloaded at: https://www.mdpi.com/article/10.3390/antibiotics15010103/s1, Supplementary File S1: Indicators for AMR surveillance costing based on FF activities; Supplementary File S2: Indicators for AMR surveillance costing based on literature review; Table S1: AMR surveillance cost by site and component; Table S2: Summary of PSA scenarios.

## Abbreviations

AMR	Antimicrobial resistance
GDP	Gross domestic product
LMIC	Low- and middle-income countries
PSA	Probabilistic sensitivity analysis
COVID-19	Coronavirus Disease 2019
OLS	Ordinary least squares
IMF	International Monetary Fund
ARIMA	Autoregressive integrated moving average
MOHP	Ministry of Health and Population
NPHL	National Public Health Laboratory
CVL	Central Veterinary Laboratory
NADIL	National Avian Disease Investigation Laboratory
Vet Lab	Veterinary Laboratory
TUTH	Tribhuvan University Teaching Hospital
DFTQC	Department of Food Technology and Quality Control
