# Supplementary material for: Understanding the Financial Implications of Antimicrobial Resistance Surveillance in Nepal: Context-Specific Evidence for Policy and Sustainable Financing Strategies
Source: Antibiotics (Basel). 2026 Jan 20;15(1):103. doi: 10.3390/antibiotics15010103 (PMC12838329; doi:10.3390/antibiotics15010103)
Supplement: Supplementary file 1 [file antibiotics-15-00103-s001.zip › Supplementary Table S1_AMR Surveillance Cost by Site and Component.DOC.pdf]

Table S1. AMR Surveillance Cost by Site and Component.

Cost-component	First 3 years (2021-2023)	Later 7 years (2024-2030)	Total
NADIL			
Total	$503,643	$1,114,071	$1,617,714
Human Resources	$113,138 (22.5%)	$301,086 (27.0%)	$414,224 (25.6%)
Allowances	$9,765 (1.9%)	$23,636 (2.1%)	$33,401 (2.1%)
Consumables	$279,441 (55.5%)	$696,313 (62.5%)	$975,753 (60.3%)
Other Direct Costs / Equipment	$101,300 (20.1%)	$93,036 (8.4%)	$194,336 (12.0%)
Vet lab			
Total	$295,138	$751,467	$1,046,606
Human Resources	$91,590 (31.0%)	$243,060 (32.3%)	$334,650 (32.0%)
Allowances	$7,322 (2.5%)	$20,478 (2.7%)	$27,800 (2.7%)
Consumables	$129,880 (44.0%)	$344,852 (45.9%)	$474,731 (45.4%)
Other Direct Costs / Equipment	$66,346 (22.5%)	$143,078 (19.0%)	$209,424 (20.0%)
Koshi			
Total	$182,326	$590,654	$772,980
Human Resources	$91,458 (50.2%)	$244,287 (41.4%)	$335,744 (43.4%)
Allowances	$253 (0.1%)	$3,856 (0.7%)	$4,110 (0.5%)
Consumables	$78,020 (42.8%)	$311,290 (52.7%)	$389,310 (50.4%)
Other Direct Costs / Equipment	$12,594 (6.9%)	$31,221 (5.3%)	$43,816 (5.7%)
TUTH			
Total	$706,783	$1,887,685	$2,594,468
Human Resources	$256,592 (36.3%)	$685,368 (36.3%)	$941,960 (36.3%)
Allowances	$603 (0.1%)	$2,858 (0.2%)	$3,461 (0.1%)
Consumables	$163,677 (23.2%)	$440,040 (23.3%)	$603,717 (23.3%)
Other Direct Costs / Equipment	$285,911 (40.5%)	$759,419 (40.2%)	$1,045,330 (40.3%)
DFTQC			
Total	$148,290	$516,002	$664,291
Human Resources	$78,511 (27.5%)	$218,639 (42.4%)	$297,149 (44.7%)
Allowances	$4,536 (1.6%)	$5,261 (1.0%)	$9,797 (1.5%)
Consumables	$18,778 (6.6%)	$244,098 (47.3%)	$262,875 (39.6%)
Other Direct Costs / Equipment	$46,466 (16.3%)	$48,004 (9.3%)	$94,469 (14.2%)
Total	$1,836,179	$4,859,879	$6,696,058

Percentages indicate each component's share of the sector total.
