# Supplementary material for: Understanding the Financial Implications of Antimicrobial Resistance Surveillance in Nepal: Context-Specific Evidence for Policy and Sustainable Financing Strategies
Source: Antibiotics (Basel). 2026 Jan 20;15(1):103. doi: 10.3390/antibiotics15010103 (PMC12838329; doi:10.3390/antibiotics15010103)
Supplement: Supplementary file 1 [file antibiotics-15-00103-s001.zip › Supplementary Table S2_Summary of PSA Scenarios.pdf]

Table S2. Summary of PSA Scenarios.

		Scenario 1	Scenario 2	Scenario 3	Scenario 4
**Number of samples**		Increase	Decrease	Increase	Decrease
**Cost-component**	**Consumables**	Unchanged from 2024 baseline	Unchanged from 2024 baseline	Proportional to sample growth from 2024 baseline	Proportional to sample decline from 2024 baseline
	**Human resources & Other direct costs / equipment**	Unchanged from 2024 baseline	Unchanged from 2024 baseline	Increasing at 1/3 sample growth rate from 2024 baseline	Decreasing at 1/3 sample growth rate from 2024 baseline
